# Caustic Injections in the Treatment of Chronic Irritation of the Bladder

**Published:** 1846-01

**Authors:** 


*Caustic Injections in the treatment of chronic irritation of the bladder.* By Dr. Debeney. (*Journ. des Connaiss & Med. et Chirurg. for April*, 1845.)

Analogy first led M. Debeney to try caustic injections for chronic irritation, or catarrh of the bladder. This mode of reasoning has led to some of the most useful discoveries in our therapeutics, and is, moreover, the most certain method of attaining to correct knowledge on many obscure points in the science of medicine. The argument here used, says M. D., is extremely simple; he first asks if the vitality of the mucous membranes is identical? and is not the membrane which lines the bladder a mucous tissue? Both those questions being answered in the affirmative, with the exception of certain parts destined to special functions, as the alimentary canal, M. D. then proceeds to detail four or five interesting observations, in which the best effects were obtained by throwing caustic injections into the bladder. Many of the cases cited, in addition to obstinate catarrh of the bladder, were also complicated with strictures, gonorrhœa, and seminal losses.

The injection used is composed of *four grammes* of crystalized nitrate of silver, dissolved in *thirty grammes* of water.—His mode of using the injection we shall give in his own language. "Having forced into the urethra, by means of a glass penis syringe, as much of the caustic fluid as possible, I close the meatus by pressing the extremity of the gland between the thumb and index finger of the left hand; then by a gradual pressure toward the root of the penis with the two first fingers of the right hand, I push the liquid so far that not a drop escapes when the pressure is suspended. It is evident that the injection must pass into the bladder, where else could it go? The pain was considerable, but quite supportable; the usual phenomena of cauterization well manifest, and at night there was slight accession of fever. Suffice it to say, that in a few days, the irritability of the urethra and bladder were allayed; less pain in passing urine, also retained much longer without inconvenience. Two or three injections were generally adequate to effect a permanent cure." Our author seems to repose unlimited confidence in the remedy above proposed, and we see no reason why it should not receive a fair trial. It must be confessed that, heretofore, chronic cystitis, irritable bladder, and all forms of cystic disease have not been generally amenable, even to the most judicious medication; let us, then, try a mode of practice, which comes highly recommended both by analogy and experience.—*New Orleans Med. Jour*,

